# More effective use of polymyxin-B hemoperfusion for nonoperation cases

**DOI:** 10.1186/cc14035

**Published:** 2014-12-03

**Authors:** M Yahata, Y Sakamoto, S Inoue, T Iwamura, R Fujita, H Koami, T Miike, H Imahase, S Narumi, A Goto, M Ohta

**Affiliations:** 1 Emergency and Critical Care Medicine, Saga University, Saga City, Japan

## Introduction

Direct hemoperfusion with polymyxin-B has been reported to improve hemodynamics in postsurgical patients. In 2012, the Japanese Guidelines for the Management of Sepsis were published and mention the efficacy of polymyxin-B direct hemoperfusion. But how to use and the target patients are varied by facilities. We investigated the effective use of polymyxin-B direct hemoperfusion in nonsurgical patients.

## Methods

We analyzed retrospectively all septic shock patients who were treated with polymyxin-B hemoperfusion between January 2008 and December 2012.We checked their mean arterial pressure (MAP), and vasopressor requirement every 30 minutes until stopping treatment.

## Results

There were 32 patients under treatment and 11 patients did not need surgical treatment. Even in the nonsurgical group, hemodynamic states and vasopressor requirement was improved after polymyxin-B hemoperfusion started. And the effects were continued over 120 minutes. A second plolymyxin-B hemoperfusion treatment underwent in nine patients. In second treatment, MAP increased in the nonsurgical group greater than in the postsurgical group.

## Conclusion

Polymyxyn-B direct hemoperfusion improves hemodynamic status even in nonsurgical patients. A second polymyxin-B direct hemoperfusion is effective especially in nonsurgical septic shock patients. And if its hemodynamic effect was significantly, long-time treatment should be considered.

